# Acknowledgement to Reviewers of Journal of Functional Morphology and Kinesiology in 2019

**DOI:** 10.3390/jfmk5010005

**Published:** 2020-01-17

**Authors:** Journal of Functional Morphology and Kinesiology Editorial Office

**Affiliations:** MDPI, St. Alban-Anlage 66, 4052 Basel, Switzerland

The editorial team greatly appreciates the reviewers who have dedicated their considerable time and expertise to the journal’s rigorous editorial process over the past 12 months, regardless of whether the papers are finally published or not. In 2019, a total of 64 papers were published in the journal, with a median time to first decision of 20.2 days and a median time from submission to publication of 37 days. The editors would like to express their sincere gratitude to the following reviewers for their generous contribution in 2019:
Albornoz-Cabello, ManuelMamen, AsgeirAlesi, MariannaMao, AihuaAlmeida Marinho, DanielMarengo, LorenzaAziz, Abdul RashidMarino, JosephBadau, DanaMario, VetranoBadicu, GeorgianMarks, RayBalague Serre, NataliaMartínez-Guardado, IsmaelBest, RussellMason, LauraBonilla, Diego A.Maugeri, GraziaBragazzi, Nicola LuigiMcGuigan, MikeBreik, OmarMeucci, MarcoBrewer, Britton W.Milardović, SlađanaBujnowska-Fedak, Maria MagdalenaMoir, GavinBuková, AlenaMolina-Hidalgo, CristinaBurns, RyanMonyeki, Kotsedi DanielBycura, DierdraMoon, SangheeCabri, JanMorris, CodyCapranica, LauraMusumeci, GiuseppeCastorina, AlessandroNohe, AnjaChan, Kuei-HuiNounagnon Frutueux, AgbanglaChauhan, SmitParra-Saldías, MaribelChrisman, MatthewPaschalis, VassilisChtourou, HamdiPatel, AmarCoco, MarinellaPavone, VitoCoratella, GiuseppePearce, Alan J.Cortis, CristinaPereira-da-Silva, LuisCosta, SebastianoPitino, DarioDe La Cuadra Blanco, CrótidaPoston, BrachDe Lira, Claudio Andre BarbosaPratas, José MariaDe Paepe, BoelQuinlan, JonathanDi Giunta, AngeloRamírez-Vélez, RobinsonDickin, ClarkReader, ChristopherD’Souza, Randall FelixRiebe, DeborahEarnest, ConradRist, BillymoEggleston, Jeffrey D.Romero Morales, CarlosFalk Neto, Joao HenriqueRose, Alister DuFernandes, John F. T.Ruas, CassioFisher, Russo, ValentinaFogelman, YacovSanders, GabeFu, XingSantos, LuisGajewski, JanSantos, RubimGarcía-García, OscarSaul, DominikGastaldi, LauraSawynok, JanaGentil, PauloSchram, BenGiannaki, ChristoforosSchulze-Tanzil, Gundula GesineGollie, JaredScifo, LidiaGómez-Andrés, DavidSeibert, Franz JosefGörgülü, RecepSerra, LídiaGuimarães-Ferreira, LucasSfondrini, Maria FrancescaHaapala, Eero A.Sormunen, JHartog, HermienSpang, ChristophHeishman, AaronStefani, LauraHernandez-Esteban, PabloStephens, RichardHerold, FabianSugiyam, KojiHofmann, PeterSun, Kai SingHolmes, Clifton J.Syed-Abdul, Majid MufaqamHosick, PeterSzychlinska, MartaHuber, RTessitore, AntonioHue, OlivierThompson, RaymondHui, JieTok, SerdarIshihara, ToruTomaz, SimoneJähn, KatharinaTomczak, AndrzejJajtner, AdamTorres-Benito, LauraJayaseelan, Dhinu J.Turkmen, MutluJin, DiUseche, Sergio A.Jin, ZhongminVanDusseldorp, TrishaKaruc, JosipVeiga, SantiagoKean, James DVillarini, AnnaKemp, JoanneVleck, VeronicaKluess, HeidiWang, YulingKoltermann, Jan JensWawrzyniak, AgataKondo, HitoshiWilk, MichalKorcz, AgataWillems, MarkKovaleski, John E.Wolanśki, WojciechKraus, KorneliusWoodfield, LorayneKumar, PRAKASHYasar, ZerbuKwak, Sang HoYeung, Andy Wai KanLavender, AndrewYu, Hsi-YuLee, KyuwanZafar, Muhammad SohailLin, Lian-yuZaffagnini, StefanoLiu, LukaiZeng, ZhengLongo, StefanoZhao, MingjunLópez-López, DanielZotti, FrancescaLorenzetti, SilvioZurita Ortega, FélixLunn, William


